# Efficient method for establishing F1 progeny from wild populations of *Anopheles* mosquitoes

**DOI:** 10.1186/s12936-017-1681-7

**Published:** 2017-01-09

**Authors:** Thiery N. Nepomichene, Lala Andrianaivolambo, Sébastien Boyer, Catherine Bourgouin

**Affiliations:** 1 Unité d’Entomologie Médicale, Institut Pasteur de Madagascar, BP 1274, Ambatofotsikely, 101 Antananarivo, Madagascar; 2 Ecole doctorale Science de la vie et de l’Environnement, Université d’Antananarivo, BP 906, Antananarivo, Madagascar; 3 Unité de Génétique fonctionnelle des Maladies Infectieuses, Institut Pasteur, 25-28 Rue du Docteur Roux, Paris, 75015 France; 4 Unité de Recherche Associée 3012, Centre National de la Recherche Scientifique, 75015 Paris, France; 5 Medical Entomology Platform, Institut Pasteur du Cambodge, 5 Preah Monivong Blvd (93), Phnom Penh, Cambodia

**Keywords:** Wild anopheles, Malaria, Madagascar, Forced-oviposition, F1 production

## Abstract

**Background:**

The changing malaria situation in Madagascar requires additional knowledge on the physiology and behaviour of local mosquito vectors. However, the absence of established colonies for several anopheline species present in Madagascar constitutes a limiting factor. To avoid labour intensive work and uncertainty for success of establishing *Anopheles* colonies from Malagasy species, field collections of blood-fed females and in-tube forced oviposition were combined to reliably produce large numbers of F1 progeny.

**Methods:**

Blood-fed females were captured in zebu stables or open zebu parks. Oviposition was induced by enclosing gravid females in eppendorf tubes as initially described for *Anopheles funestus*. The effect of cold anaesthesia on inducing in-tube forced oviposition and on egg yield was assessed for five *Anopheles* species, namely *Anopheles coustani*, *An. funestus*, *Anopheles mascarensis*, *Anopheles arabiensis* and *Anopheles squamosus*. The production of eggs from in-tube forced oviposition and standard egg laying in cages was compared.

**Results:**

For the five anopheline species studied, the in-tube forced oviposition method had different efficacy ranging from 35.6 to 71.1% females willing to lay eggs in tubes. Interestingly, prior anaesthesia increased significantly the proportion of ovipositing females for *An. mascarensis*. Prior anaesthesia has a marginal effect on the number of eggs produced. However, the overall yield in eggs collected using the in-tube forced oviposition method largely exceeds the number of eggs that can be produced by females free to oviposit in cages.

**Conclusion:**

The efficiency of the method allowed the production of F1 progeny in numbers sufficiently large for developing detailed analyses of the five species tested, including behavioural studies, insecticide resistance assessment and molecular characterization, as well as vector competence studies. It should be applicable to other anopheline species difficult to colonize.

## Background

Mosquitoes constitute a large group of arthropod vectors of pathogens. Among them the *Anopheles* genus includes all known vectors of malaria parasites infecting mammals. One feature of *Anopheles* mosquitoes as malaria vectors is that they exhibit a highly specific geographic distribution [1]. As a consequence, each malaria endemic country harbours its own set of major and/or potential secondary anopheline vectors. This is in sharp contrast with the large geographic distribution of arbovirus culicine vectors, exemplified by *Aedes aegypti*, which can be found in most tropical areas, and *Aedes albopictus*, which expanded its distribution range from tropical to more continental areas in the last two decades [2].

Among the ~60 known human malaria vectors, very few have been easily colonized, limiting in-depth study of their biological characteristics [3]. Over many years scientists have developed strategies for colonizing several anopheline species in the laboratory with successes and failures. A common feature of several anopheline mosquitoes is eurygamy, which hampers efficient mating in a confined environment. To counteract the absence of free mating in cages, the technique of forced mating was developed and shown to work for several species [4, 5], *Anopheles dirus* being among the best-known examples. This mating technique allowed the establishment of optimized production of some anopheline species at the cost of time. Alternatively, long-term efforts led to the selection of individuals that accept mating in a confined environment and to the establishment of so-called free-mating colonies [6]. For other mosquito species introducing tricks such as a stroboscopic light has shown to be effective in inducing mating [7–9]. However, these tricks turn out not to be efficient for establishing colonies from every single anopheline species. Even in situations where rearing success has been reported, the reasons for success are often obscure and not repeatable outside the successful laboratory. Establishment of *Anopheles funestus* colonies was such an example [10, 11].

In Madagascar, three anopheline species (*Anopheles gambiae*, *Anopheles arabiensis*, *An. funestus)* are considered major vector species of malaria. *Anopheles mascarensis*, an endemic species, and *Anopheles merus* have been identified as secondary vectors of local importance [12–17]. Due to their high abundance, two other species, *Anopheles squamosus* and *Anopheles coustani*, are suspected to be involved in residual malaria transmission, in places where the major vectors are of low abundance. Indeed, *An. coustani* has recently been described as a potential secondary malaria vector in Madagascar [18]. Whereas *An. gambiae* and *An. arabiensis* can be easily colonized, none of the other species have been successfully colonized yet, despite reports on existing current or past colonies of these species [10, 11, 19].

The malaria transmission pattern is currently changing in Madagascar with an epidemic situation for the past few years [20]. Among several other causes, there is suspicion of increased transmission by *An. mascarensis* and *An. coustani*. The changing malaria situation in Madagascar advocates for the urgent need of gaining additional knowledge on these two species. To avoid labour intensive work and uncertainty for success of establishing *Anopheles* colonies from Malagasy species including *An. mascarensis* and *An. coustani*, field collection of blood-fed females was combined with in-tube forced oviposition to reliably produce F1 progenies. The efficiency of the method allowed the production of F1 in numbers sufficiently large to permit detailed analyses of those species, including behavioural studies, insecticide resistance assessment and molecular characterization, as well as vector competence studies. The in-tube forced oviposition method was first reported by Morgan et al. [21], successfully producing F1 from *An. funestus* and later included into the MR4 manual 2014 [22].

To the original method an additional step was introduced, which turns out to be highly efficient for some species for increasing the number of females willing to lay eggs and subsequently increasing the number of the F1 progeny. Herein is presented a detailed analysis of the benefit of this strategy for producing F1 from four *Anopheles* species encountered throughout Africa (*An. arabiensis, An. funestus, An. coustani* and *An. squamosus*) and one Malagasy species *An. mascarensis*. The demonstration that this method, initially developed for *An. funestus*, is easily applicable to additional anopheline species should facilitate a better characterization of malaria vectors from different countries, for which no sustainable colonies exist yet.

## Methods

### Mosquito sampling

**Fig. 1:**
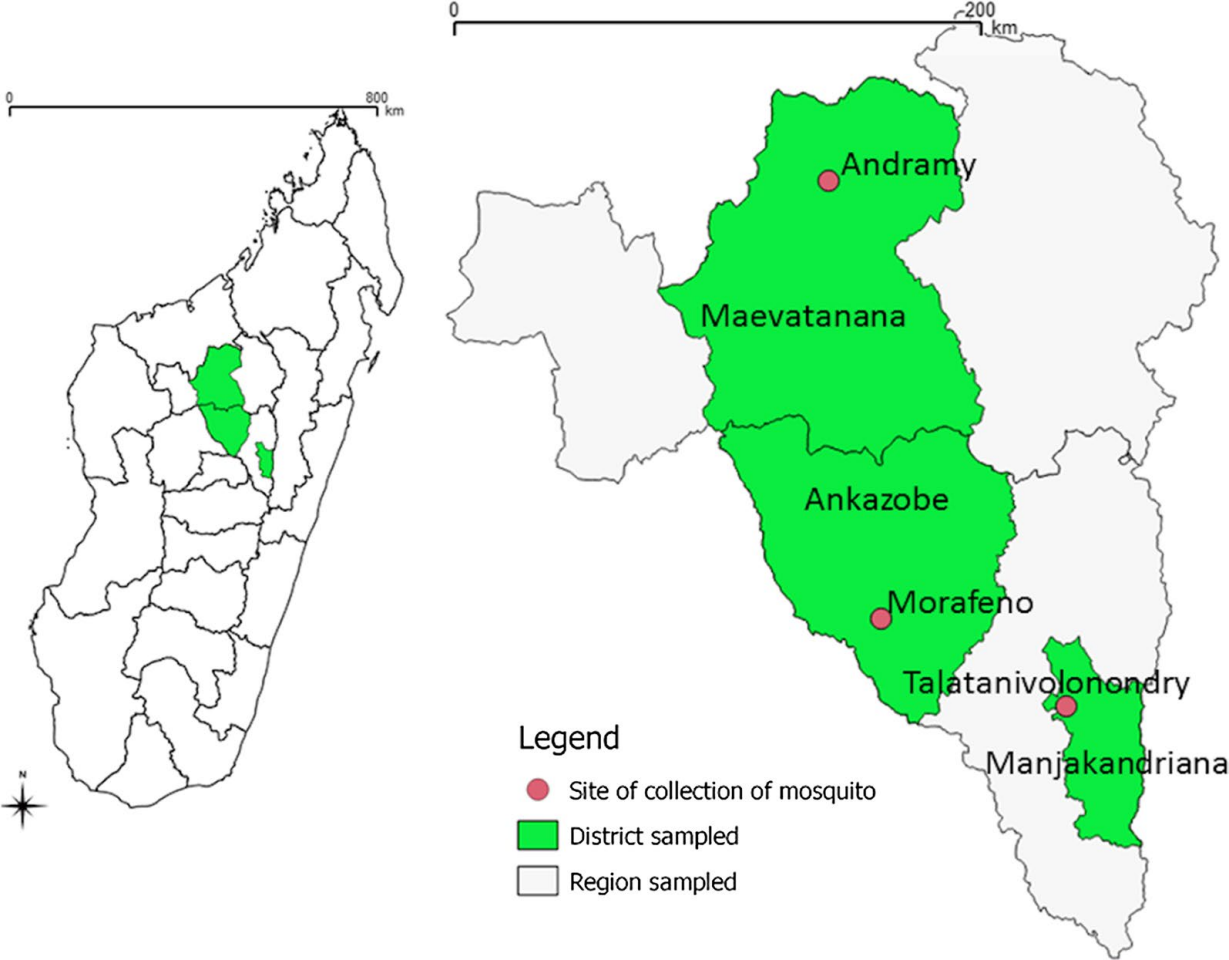
Location of the sites for mosquito collections. The *left panel* is a schematic drawing of Madagascar. The *right panel* is an enlargement of the region where mosquitoes were collected with villages highlighted in* red*

Adult female mosquitoes were collected in three villages namely Andramy (S16°54′37.56″; EO46°52′17.54″) in the district of Maevatanana, Morafeno (S18°24′13″; EO 47° 03′ 03″) in the district of Ankazobe, and Talatavolonondry (S18°41′56.59″; EO47°40′40.66″) in the district of Manjakandriana (Fig. 1). Morafeno and Talatavolonondry are located in the Central Highlands of Madagascar. These sites are characterized by the dominance of rice fields as breeding sites for mosquitoes. Andramy is located in the Western region of Madagascar, where the major mosquito breeding sites are small water collections along riverbanks. In each location, the rainy season begins in November and lasts until May. Sampling was performed in April 2014, January 2015 and April 2016 for Morafeno; in April 2014 for Andramy, and in July and November 2014 for Talatavolonondry.

### Mosquito collection methods

**Fig. 2:**
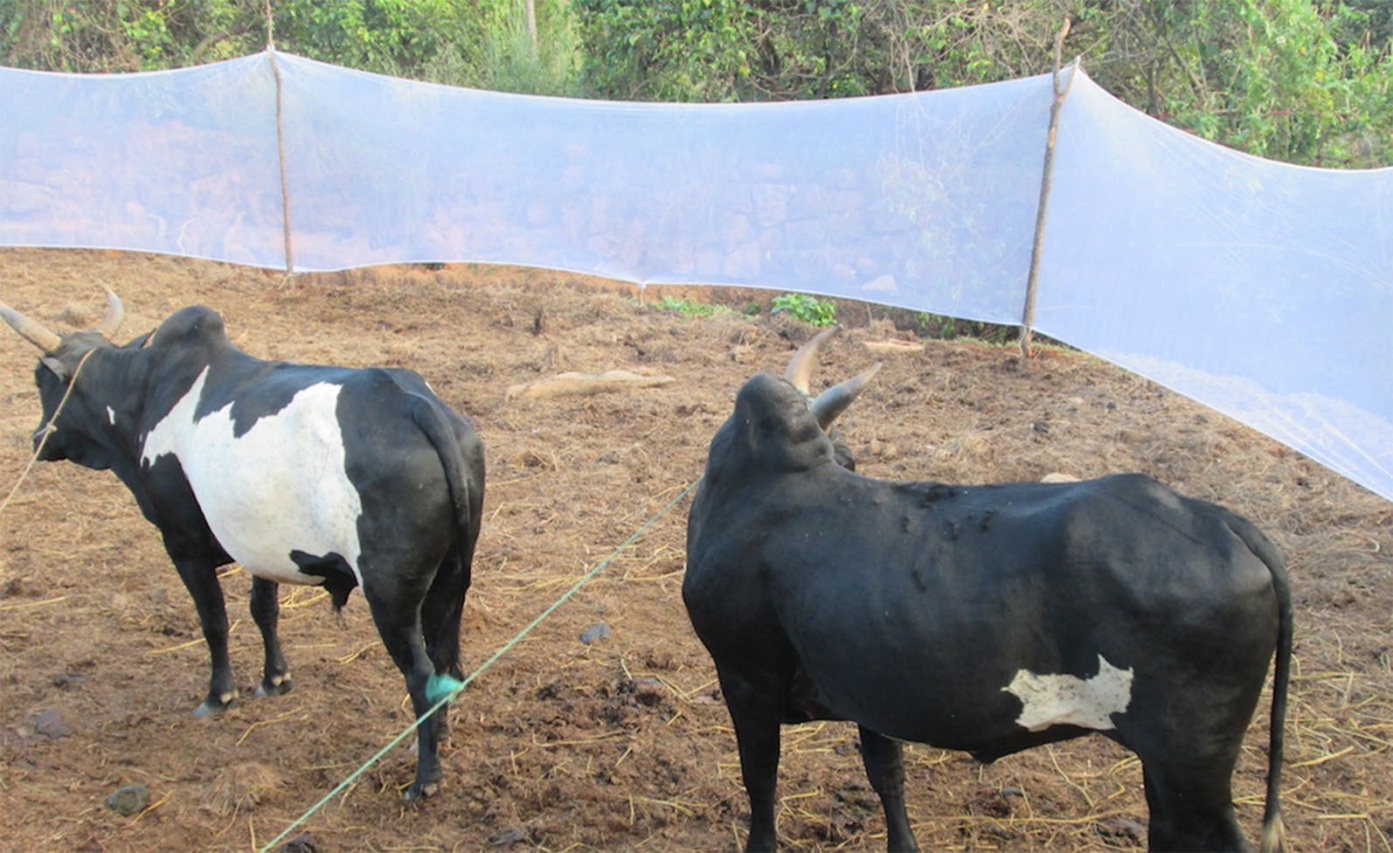
Setting for trapping blood-fed mosquitoes in an open zebu park. A wide mosquito net is placed around the park, leaving one side open for mosquitoes to enter the site. Mosquitoes trapped by the net after feeding on zebus are caught one by one, using a mouth aspirator

Blood-fed female mosquitoes were captured by two methods, either resting in cattle stables or trapped by a net placed around an open zebu park, as depicted in Fig. 2, following a method described by Fara Nantenaina Raharimalala [23]. In this case, female mosquitoes had recently blood-fed on zebus. For both methods, females were collected using a mouth aspirator and transferred into a large mosquito cage. Collections in the stables were performed in the morning between 7:00 and 9:00 h, whereas collections in the zebu parks were performed at night (19:00, 12:00 am and 02:00). Captured mosquitoes were given access to a 10% sucrose solution by placing a moistened cotton ball on the top of the cage. They were transported within 2 days to the laboratory in Antananarivo and sorted by species using morphological criteria (Fontenille, unpublished 1989, [24]).

### Egg collection

**Fig. 3:**
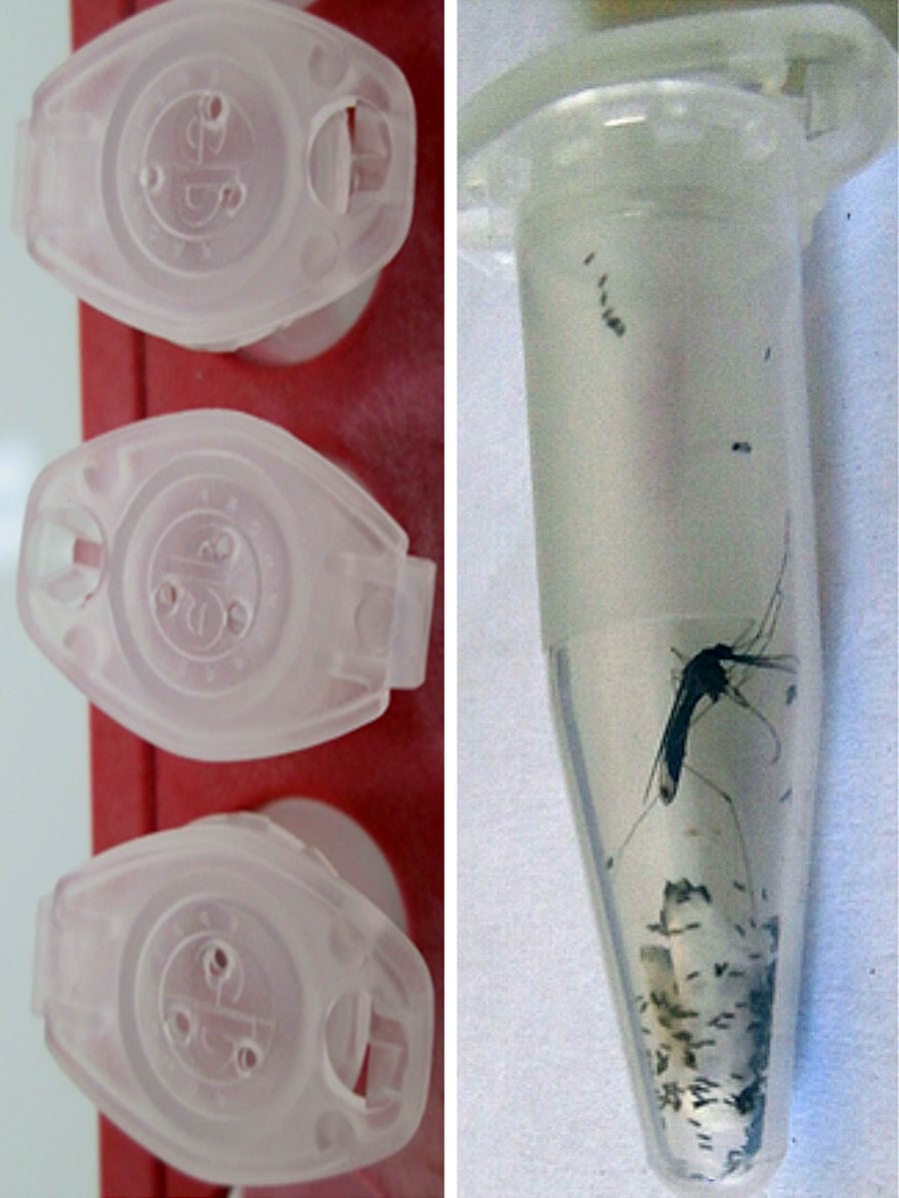
In-tube forced oviposition. Gravid females are introduced into an Eppendorf tube, which top contains three holes. Female lay eggs on a small piece of moisten filter paper placed at the *bottom* of the tube

Female mosquitoes were maintained at 27 °C ± 2, 75% RH with free access to 10% sucrose until they became fully gravid, five days later. Mosquito eggs were collected by two methods: in tube or in cage. The in-tube collection method, named hereafter in-tube forced oviposition, is based on the method described by Morgan et al. [21]. Briefly, females are individually placed inside an Eppendorf® tube with a moistened 1 cm^2^ piece of filter paper placed at the bottom, and the cap pierced with three holes (Fig. 3). To introduce the females inside the tube, half of them, randomly chosen, were first cold anaesthetized and transferred inside the tube using forceps; from the remaining half, females were randomly captured one by one from within the cage, using the above described modified Eppendorf tubes. Mosquitoes were observed daily for egg laying and survival for 5 days (Fig. 3). Oviposited eggs from each female were counted after removal of the filter paper. For egg collection in cages, a Petri dish containing moistened filter paper was used, except for *An. funestus* for which we used a container with black sides. Eggs were count and removed each day for up to five days and the average egg number per female was calculated by dividing the total number of eggs by the number of females in the cage.

### Mosquito rearing

Eggs were transferred into a rearing pan containing dechlorinated tap water. L1 and L2 larvae were fed with Tetramin™ baby fish food and L3 and L4 larvae either with cat or mice finely ground food. Water from each larval pan was changed every other day. The F1 adults were mixed in cages for subsequent experiments.

### Data analyses

Chi square test was used for comparing the proportion of female mosquitoes that laid eggs by in-tube forced oviposition with or without prior cold-anaesthesia. Wilcoxon test was used for comparing egg numbers produced by those females. These tests were performed using R Core Team (2013).

### Results and discussion

Blood-fed *Anopheles* females (n = 1026) were captured over six sampling periods from April 2014 until April 2016 in three different locations. Most specimens were trapped using a large mosquito net set up on one side of a zebu park (see Fig. 2 and “Methods” section) from 7 pm till 2 am and collected using mouth aspirators. Resting mosquitoes were also collected within zebu stables in the morning. For each harvest, all captured mosquitoes were placed inside a single large cage before being morphologically identified and sorted on the next day. Fully fed females from each species were maintained in the insectary for an additional three days until fully gravid, with free access to only sugar solution. At that stage, females were individually placed into 1.5 ml Eppendorf^**®**^ tubes prepared as described in “Methods” section and observed over 5 days to determine their ability to lay eggs under these conditions. To control the gravid state of the females, a proportion of females were anaesthetized on ice and observed under a binocular microscope before being placed in tubes.

**Fig. 4:**
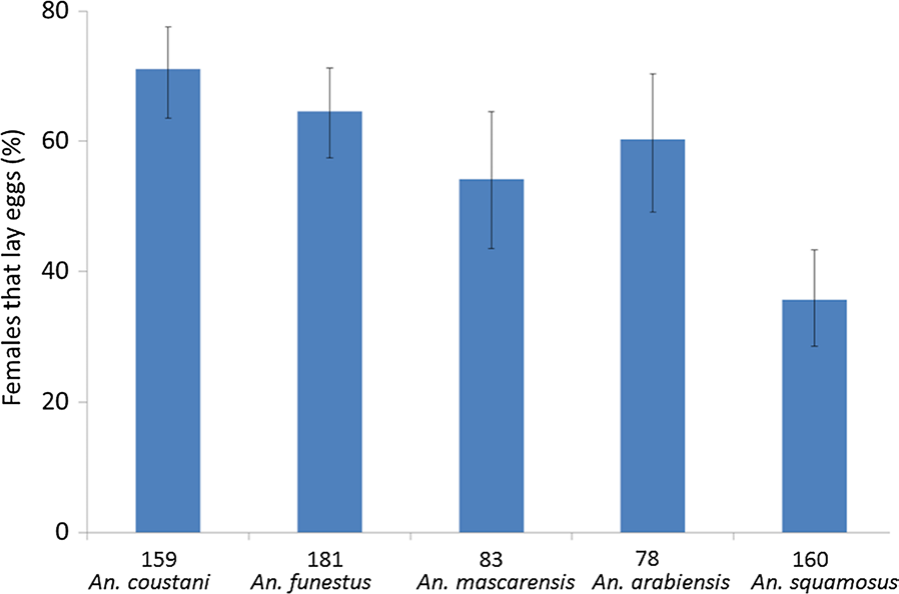
In-tube forced oviposition efficiency. Mosquito females were place in tube 5 days after capture. Proportion of female laying eggs in tube was recorded over the next 5 days. The number of females used for each species is indicated underneath each plot. These numbers include females anaesthetized and non-anaesthetized prior oviposition.* Bars* represent the 95% confidence interval for proportions [26, 27]

**Fig. 5:**
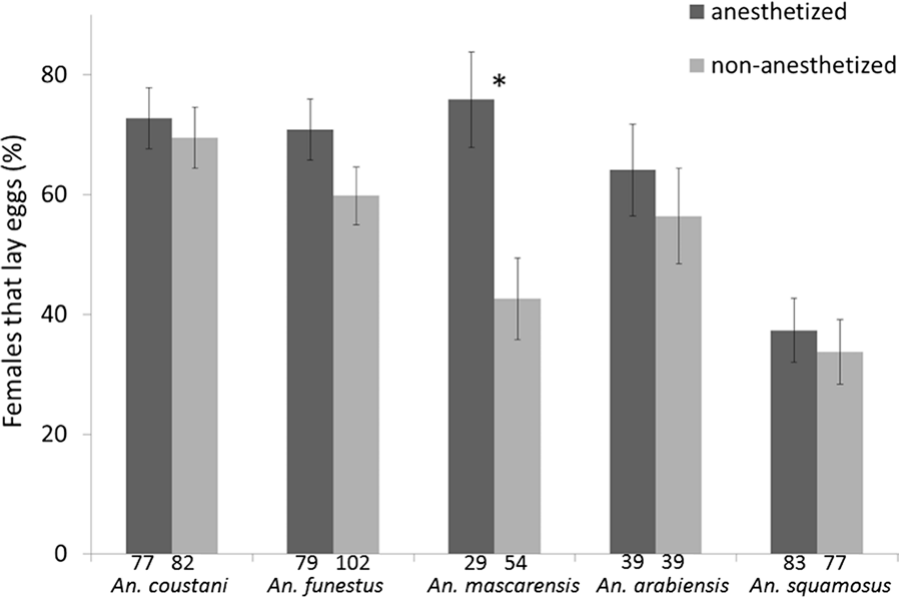
Effect of prior anaesthesia on forced oviposition efficiency. The *graph* compares the proportion of females laying eggs in tubes, whether anaesthetized (*dark grey*) or not anaesthetized (*light grey*) prior oviposition. Samples sizes used for each species are indicated underneath each plot.* Bars* represent the 95% confidence interval for proportions. *Asterisk* indicates significant difference between anaesthetized and non-anaesthetized (Chi square test)

All anaesthetized females observed under the binocular microscope were fully gravid confirming that both methods used to capture blood-fed females were efficient. The proportion of females that would lay eggs while maintained enclosed into Eppendorf^**®**^ tubes was then determined. As presented in Fig. 4, the in-tube forced oviposition method was successful for all tested species although with variable efficiency ranging from 71.07% for *An. coustani* down to 35.63% for *An. squamosus*. Interestingly, it was observed that some batches of females that were cold-anaesthetized before being held in tubes tend to be more willing to lay eggs than the ones that were not. As presented in Fig. 5, a significant difference was observed for *An. mascarensis* between cold anaesthetized and non-anaesthetized females (X-squared = 7.126, df = 1, p = 0.008). A slight effect was also observed for the other species, but the differences between cold anaesthetized females and non-anaesthetized ones were not significant (X-squared = 0.074, df = 1, p = 0.786; X-squared = 1.932, df = 1, p = 0.165; X-squared = 0.214, df = 1, p = 0.644 X-squared = 0.95, df = 1, p = 0.758 for *An. coustani, An. funestus, An. arabiensis and An. squamosus*, respectively).

**Fig. 6:**
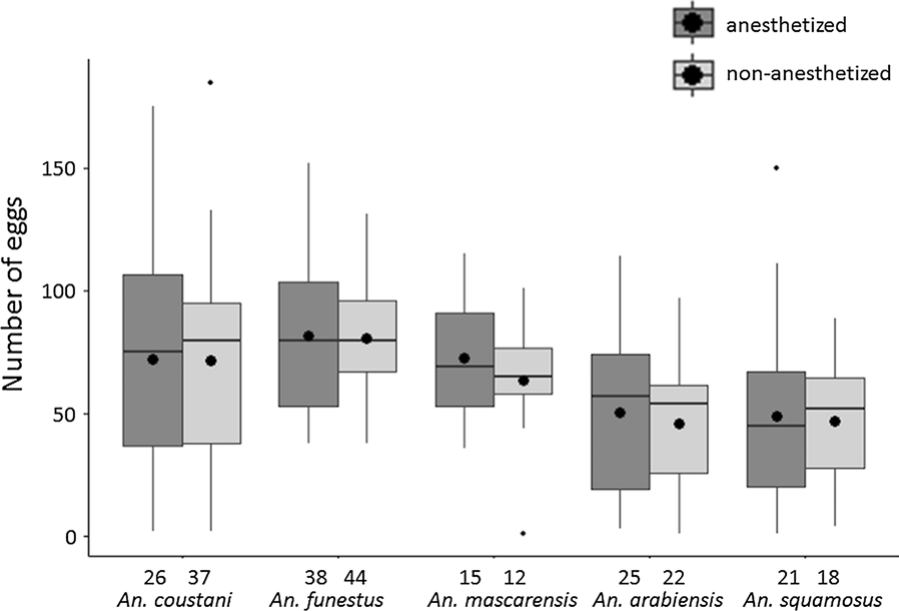
Egg production by females forced to oviposit. *Box* plot representation of the egg numbers produced by each female willing to oviposit in tubes, either anaesthetized (*dark grey*) or not anaesthetized (*light grey*) prior to oviposition. Samples sizes used for each species are indicated underneath each plot. *Black dots* mean values

Next, the number of eggs produced by each female that laid eggs by forced oviposition in tubes was quantified. Most of those females produced a reasonable number of eggs with mean number ranging from 45.86 eggs for non-anaesthetized *An. arabiensis* to 81.71 eggs for cold-anaesthetized *An. funestus* (Fig. 6). For each species, there was no significant differences among females whether they were cold-anaesthetized prior to in-tube forced oviposition or not, although *An. mascarensis* females tended to lay more eggs after cold anaesthesia ((W = 477.5, p = 0.599; W = 829, p = 0.951; W = 107.5, p = 0.406; W = 312, p = 0.436; W = 182.5, p = 0.865 for *An. coustani, An. funestus, An. mascarensis, An. arabiensis and An. squamosus,* respectively). Interestingly, crossing the percentage of females that laid eggs with the egg yield per female indicates that the in-tube forced-oviposition method provide an efficient way to produce a large number of F1 progeny from all tested species. Indeed, based on our results, one can expect for 100 females captured as blood-fed females, a minimum of roughly 1700 eggs for *An. squamosus* (Table 1). This number can reach more than 5000 eggs for *An. coustani*, *An. funestus* and *An. mascarensis* if females are cold anaesthetized prior to in-tube forced oviposition. Producing eggs is not enough for producing F1 progeny suitable for biological studies. Therefore, we verified that each egg batch series indeed produced a large adult F1 population, using standard anopheline procedures for larval stage rearing.

**Table 1:** Estimation of egg production for 100 females by in-tube forced oviposition

Species	Proportion of females laying eggs in tubes (%)	Egg number per female laying eggs		Yield for 100 females		
		Mean	SD	Egg number	SD	Range
*An. coustani*	71.07	71.83	43.96	5106.83	3124.24	[1983; 8231]
*An. funestus*	64.64	81.17	26.24	5246.95	1696.15	[3551; 6943]
*An. mascarensis*	54.22	68.63	24.53	3720.88	1330.02	[2391; 5051]
*An. arabiensis*	60.26	48.4	28.59	2916.67	1722.83	[1194; 4640]
*An. squamosus*	35.63	48.13	32.78	1714.57	1167.95	[547; 2883]

The egg mean number corresponds to pooled data from anaesthetized and non-anaesthetized females; *SD* standard deviation. The yield standard deviation was calculated as follows: mean egg number per female SD × Proportion %

The generation time from egg to adult was around 15–17 days for *An. funestus*, confirming the observation of Cuamba et al. [25]. It was similar for *An. arabiensis* and *An. mascarensis*, but in the range of 21 days for both *An. coustani* and *An. squamosus.*

**Fig. 7:**
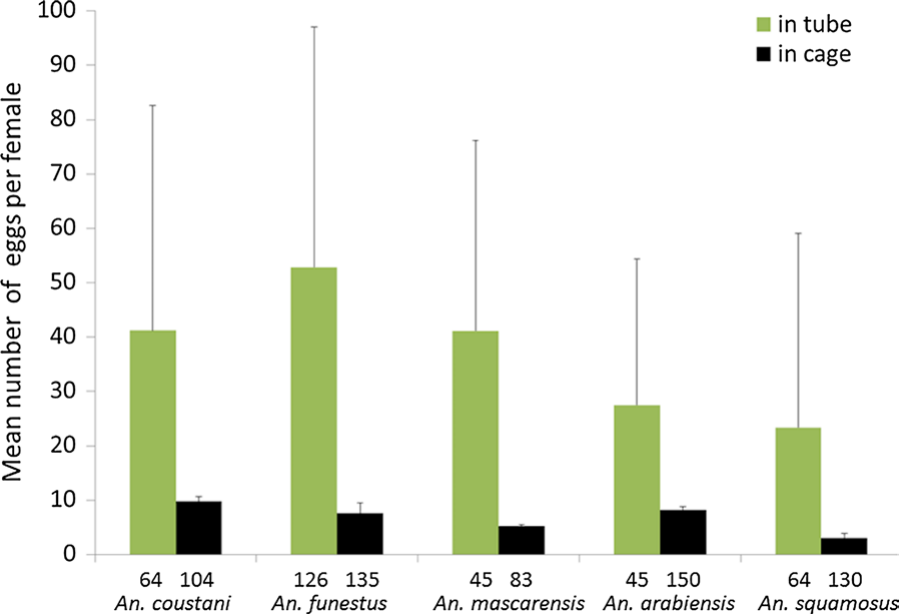
Egg production by in-tube forced oviposition versus free oviposition in cages. Gravid females were either forced to oviposit in tubes or let free to oviposit in cages. The total number of eggs collected per condition and per species was normalized to the total number of females used. This includes females that did not lay eggs in the in-tube forced oviposition samples. The sample size is indicated underneath each plot. For in-tube forced oviposition, bars represent standard deviation of the egg mean number per female, while for free oviposition in cages,* bars* represent the standard deviation among cage replicates

To further confirm that the in-tube forced oviposition method offers considerable advantages, the number of eggs produced by females free to oviposit in cages was compared to the number of eggs produced by females forced to oviposit in tubes. For this experiment, a single field collection of mosquitoes was used; mosquitoes were randomly assigned to forced-oviposition or placed in two independent cages for all species but *An. funestus*, for which three cages were set up. Egg collection devices were made of damped filter paper in a petri dish, except for *An. funestus* where damped filter paper was placed in a dark bowl, as commonly recommended for *An. funestus*. As among the females in the cage one cannot tell which one laid eggs or not, the total number of eggs collected in cage was compared to the total number of eggs from in-tube females whether they had or not laid eggs. Results presented in Fig. 7 clearly show that the in-tube forced oviposition is highly superior to free oviposition in rearing cages for collecting large number of eggs and subsequent production of F1 progeny from captured blood-fed females. This is valid for all species tested in this report with an increase in egg production varying from 234% (*An. arabiensis)* to 678% (*An. mascarensis*) as reported in Table 2.

**Table 2:** Egg collection increase by in-tube forced oviposition

Species	Mean egg number per female		Increase % in egg yield
	In-tube forced oviposition	In-cage free oviposition	
*An. coustani*	41.18	9.78	321
*An. funestus*	52.82	7.58	597
*An. mascarensis*	41.17	5.19	693
*An.arabiensis*	27.48	8.23	234
*An. squamosus*	23.35	3.00	678

The mean egg number takes into account all females put in tubes or in cages from a single field collection as plotted in Fig. 7. Increase % in egg yield was calculated as follows: 100 × (mean number in tube-mean number in cage)/mean number in cage

## Conclusions

This work demonstrates that the in-tube forced oviposition method initially developed for *An. funestus* applies efficiently to other anopheline species. For *An. funestus* the same efficiency as the one reported by Morgan et al. was observed [21]. Furthermore, inclusion of a cold anaesthesia step to *An. mascarensis* increases significantly the number of females that oviposit in tubes. Although this does not significantly apply to the other mosquito species tested, this might be worth trying for other anopheline species. Overall the comparison of egg yield between in-tube forced oviposition and free oviposition in cages clearly shows that establishing large F1 populations can be easily achieved using the in-tube forced oviposition method and should be favoured for conducting detailed analyses on behavioural studies, insecticide resistance assessment and molecular characterization or vector competence studies.
